# Primary Sternal Tubercular Osteomyelitis: A Rare Cause of Chronic Chest Pain

**DOI:** 10.7759/cureus.13959

**Published:** 2021-03-17

**Authors:** Hani Saif

**Affiliations:** 1 Obstetrics and Gynecology, Niger Central Hospital, Niamey, NER

**Keywords:** tuberculosis, osteomyelitis, chest pain

## Abstract

Sternal osteomyelitis is a rare clinical condition that occasionally develops when there is an adjacent focus of infection. However, primary sternal tubercular osteomyelitis is an extremely rare form. We describe the case of a 52-year-old Indian male who presented with a two-year history of central chest pain. The pain was not related to exercise, meals, or respiration. There was no history of cough, fever, or weight loss. On examination, there was tenderness over the sternum and the patient was diagnosed as having costochondritis. However, the patient's condition did not improve and he developed erythematous skin changes over the sternum. A computed tomography scan of the chest demonstrated destructive sternal lesions. Subsequently, a biopsy was taken which revealed the diagnosis of tuberculosis. The patient was managed by anti-tubercular therapy and had complete resolution of symptoms. Clinicians should keep this condition in the differential diagnosis of chronic chest pain mimicking the diagnosis of costochondritis, particularly in patients from endemic areas.

## Introduction

Sternal osteomyelitis refers to an infection of the marrow of the sternal bone, which may be primary or secondary in nature. In secondary osteomyelitis, there is an adjacent focus of infection, which usually develops in cardiac surgeries, intravenous drug users, and immunocompromised patients. However, in primary osteomyelitis, which is less common, there is no predisposing factor [1]. Given the broad differential diagnosis of chest pain, sternal osteomyelitis is not often considered due to its rarity. Missed diagnosis of sternal osteomyelitis is associated with increased morbidity [2]. Here, we present the case of a middle-aged man who presented with isolated chest pain and was found to have primary sternal osteomyelitis due to tuberculosis.

## Case presentation

A 52-year-old Indian man presented with a two-year history of non-specific central chest pain. His pain started gradually and had been increasing in severity. It started to interrupt his daily activities. The pain was not related to exercise, meals, or respiration. His pain was not relieved by simple analgesics. The patient reported no history of trauma. The pain was not associated with cough, fever, anorexia, or weight loss. Otherwise, the patient was healthy and his past medical history is unremarkable.

On examination, his vital signs were within the normal limits. Examination of the chest revealed central tenderness over the sternum. Lung auscultation revealed normal vesicular breathing throughout both lung fields with no added sounds. Cardiac sounds were normal. The frontal chest radiograph showed clear lung fields. A 12-lead electrocardiograph demonstrated a normal sinus rhythm. In light of these findings, the patient was diagnosed as having costochondritis. He was prescribed non-steroidal anti-inflammatory agents and was given a follow-up appointment.

After two weeks, the patient presented again with no improvement in his pain. However, erythematous changes over the sternum were observed. Routine laboratory investigation, including hematological, liver, renal function tests, and inflammatory markers were within normal limits. Considering the recent changes and the long history of pain, the patient was referred to have a computed tomography scan of the chest.

**Figure 1:**
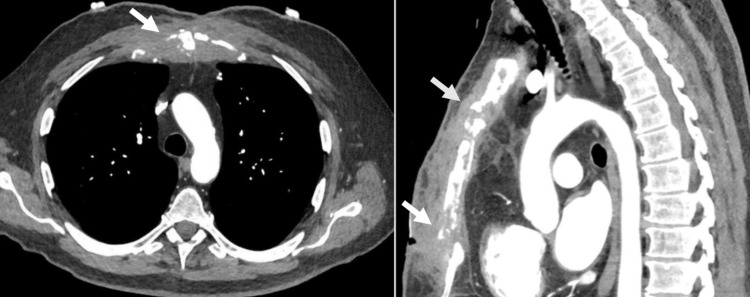
Computed Tomography of Thorax Axial and sagittal computed tomography images of the thorax showing destructive osseous lesions of the sternum (arrow)

The computed tomography scan revealed extensive destructive lesions in the sternal bone, suggestive of the diagnosis of sternal osteomyelitis (Figure 1). Subsequently, a specimen was obtained from the sternum for culture and histopathological examinations. The biopsy revealed the growth of Mycobacterium tuberculosis.

Considering these findings, the patient was referred to the infectious disease team for management. He was commenced on anti-tubercular medications, including rifampin, isoniazid, pyrazinamide, and ethambutol, for 12 months. The patient was followed-up with frequently during the course of treatment and improvement in his symptoms was evident. After the completion of the treatment course, the patient had a complete resolution of his symptoms.

## Discussion

We described the case of primary osteomyelitis of the sternum due to tuberculosis. Interestingly, this was the only presentation of his tuberculosis disease. Tuberculosis is a global healthcare problem, particularly in developing countries. Tuberculosis can present with pulmonary and extrapulmonary manifestations [3].

Tuberculosis may involve any organ in the body by secondary infection. The extra-pulmonary manifestation of tuberculosis, as in the present case, accounts for 30% of all tuberculosis cases [4]. However, primary osteomyelitis of tuberculosis is exceedingly rare with few reported cases. Hence, the diagnosis is not uncommonly missed.

Given the rarity of the condition, there are no consensus guidelines on the management of primary sternal osteomyelitis due to tuberculosis. Some physicians advise that surgical debridement is essential to prevent a recurrence. However, anti-tubercular medications remain the mainstay of treatment [4].

## Conclusions

Primary sternal osteomyelitis is an extremely rare presentation of tuberculosis. Clinicians should keep this condition in the differential diagnosis of chronic chest pain mimicking the diagnosis of costochondritis, particularly in patients from an endemic area. In the present case, medical therapy in the form of anti-tubercular medications was successful in relieving the symptoms of the patient.
